# Enhanced 3-Sulfanylhexan-1-ol Production in Sequential Mixed Fermentation with *Torulaspora delbrueckii*/*Saccharomyces cerevisiae* Reveals a Situation of Synergistic Interaction between Two Industrial Strains

**DOI:** 10.3389/fmicb.2016.00293

**Published:** 2016-03-15

**Authors:** Philippe Renault, Joana Coulon, Virginie Moine, Cécile Thibon, Marina Bely

**Affiliations:** ^1^Unité de Recherche Œnologie, EA 4577, Institut des Sciences de la Vigne et du Vin, University of BordeauxVillenave d’Ornon, France; ^2^BioLaffortBordeaux, France; ^3^Unité de Recherche Œnologie, USC 1366, Institut des Sciences de la Vigne et du Vin, Institut National de la Recherche AgronomiqueVillenave d’Ornon, France

**Keywords:** non-*Saccharomyces*, *Torulaspora delbrueckii*, wine, fermentation, mixed inoculation, volatile thiols, aroma precursors

## Abstract

The aim of this work was to study the volatile thiol productions of two industrial strains of *Torulaspora delbrueckii* and *Saccharomyces cerevisiae* during alcoholic fermentation (AF) of Sauvignon Blanc must. In order to evaluate the influence of the inoculation procedure, sequential and simultaneous mixed cultures were carried out and compared to pure cultures of *T. delbrueckii* and *S. cerevisiae*. The results confirmed the inability of *T. delbrueckii* to release 4-methyl-4-sulfanylpentan-2-one (4MSP) and its low capacity to produce 3-sulfanylhexyl acetate (3SHA), as already reported in previous studies. A synergistic interaction was observed between the two species, resulting in higher levels of 3SH (3-sulfanylhexan-1-ol) and its acetate when *S. cerevisiae* was inoculated 24 h after *T. delbrueckii*, compared to the pure cultures. To elucidate the nature of the interactions between these two species, the yeast population kinetics were examined and monitored, as well as the production of 3SH, its acetate and their related non-odorous precursors: Glut-3SH (glutathionylated conjugate precursor) and Cys-3SH (cysteinylated conjugate precursor). For the first time, it was suggested that, unlike *S. cerevisiae*, which is able to metabolize the two precursor forms, *T. delbrueckii* was only able to metabolize the glutathionylated precursor. Consequently, the presence of *T. delbrueckii* during mixed fermentation led to an increase in Glut-3SH degradation and Cys-3SH production. This overproduction was dependent on the *T. delbrueckii* biomass. In sequential culture, thus favoring *T. delbrueckii* development, the higher availability of Cys-3SH throughout AF resulted in more abundant 3SH and 3SHA production by *S. cerevisiae.*

## Introduction

Volatile thiols are powerful aromatic compounds that contribute to the fruity notes of many white wines, especially Sauvignon Blanc. The three most important thiols in Sauvignon Blanc aroma are considered to be 3-sulfanylhexan-1-ol (3SH, formerly known as 3MH; Tominaga et al., 1998a), its acetate, 3-sulfanylhexyl acetate (3SHA, formerly known as 3MHA; Tominaga et al., 1996), and 4-methyl-4-sulfanylpentan-2-one (4MSP, formerly known as 4MMP; Darriet et al., 1995; Tominaga et al., 1996, 1998a). Descriptors such as box tree and broom for 4MSP and grapefruit/passion fruit for 3SH match the occurrence of these compounds in box tree and yellow passion fruit, respectively (Tominaga and Dubourdieu, 1997, 2000). Due to their low perception thresholds (a few ng/L), they contribute significantly to the aroma profile of many wines (Roland et al., 2011 and cited references).

The release of volatile thiols by *Saccharomyces cerevisiae* yeast during alcoholic fermentation (AF), now relatively well described, results from the biotransformation of non-odorous precursors present in grapes (Tominaga, 1998; Marullo and Dubourdieu, 2010). 4MSP and 3SH are produced from cysteinylated (Cys-4MSP, Cys-3SH) and glutathionylated (Glut-4MSP, Glut-3SH) conjugates by yeast β-lyase cleavage (Darriet et al., 1995; Tominaga et al., 1998b; Peyrot des Gachons et al., 2002; Subileau et al., 2008a; Fedrizzi et al., 2009; Roland et al., 2011; Coetzee and du Toit, 2012). The biotransformation of these precursors by yeast involves their uptake through the membrane, followed by cleavage into their corresponding aromas (for a review, see Coetzee and du Toit, 2012). Concerning 3SH, in *S. cerevisiae*, the cysteinylated precursor form is taken up by amino acid transporters, such as Gap1p (Subileau et al., 2008b), while the glutathionylated form is assimilated through the Opt1p GSH transporter (Subileau et al., 2008a). Once transported into the cytoplasm, these precursors are transformed by α,β-elimination, catalyzed by β-lyases (Howell et al., 2005; Thibon et al., 2008; Holt et al., 2011; Roncoroni et al., 2011; Cordente et al., 2015). However, biotransformation rates by *S. cerevisiae* are low, with calculated yields ranging from <1% to about 5% (Murat et al., 2001; Dubourdieu et al., 2006; Grant-Preece et al., 2010; Kobayashi et al., 2010; Winter et al., 2011). 3SHA is produced after 3SH release by alcohol acetyltransferase, encoded by the ATF1 gene in *S. cerevisiae* (Swiegers et al., 2006). The final concentration of 3SHA depends on the activity balance between alcohol acetyltransferase (promoting esterification of the corresponding alcohol) and esterase (promoting its hydrolysis), encoded by the IAH1 gene (Coetzee and du Toit, 2012).

In recent years, several authors have highlighted the positive contribution of non-*Saccharomyces* yeasts to the analytical and sensory composition of wine, leading to the commercialisation of certain non-conventional yeasts. This is the case of the *Torulaspora delbrueckii* species, now available as an active dry yeast. Indeed, this species has been described as having a positive impact on the organoleptic quality of wines, due to its low production of compounds such as acetic acid, ethyl acetate, acetaldehyde, acetoin, hydrogen sulfide and volatile phenols, hence minimizing off-flavors (Cabrera et al., 1988; Herraiz et al., 1990; Martinez et al., 1990; Ciani and Picciotti, 1995; Ciani and Maccarelli, 1998; Shinohara et al., 2000; Plata et al., 2003; Renault et al., 2009). A strong β-glucosidase activity, which enhances wine aroma by hydrolysing terpenyl-glycosides, was also described in several *T. delbrueckii* strains (King and Richard Dickinson, 2000; Hernandez-Orte et al., 2008; Comitini et al., 2011; Azzolini et al., 2012). Moreover, overall, *T. delbrueckii* alone produced lower quantities of esters than *S. cerevisiae* (Viana et al., 2008; Sadoudi et al., 2012) but a few minor esters (ethyl propanoate, ethyl isobutanoate, and ethyl dihydrocinnamate) were produced in larger concentrations, which had a positive organoleptic impact on the wine (Renault et al., 2015).

Despite a good ethanol production (up to 11% vol ethanol) compared to other non-*Saccharomyces* yeasts (Cabrera et al., 1988; Herraiz et al., 1990; Ciani and Picciotti, 1995; Ciani and Maccarelli, 1998; Renault et al., 2009; Velázquez et al., 2015), *T. delbrueckii* alone cannot complete AF under winemaking conditions. *T. delbrueckii/S. cerevisiae* multi starters have thus been proposed to modulate wine flavor and properties and to ensure complete AF. An increasing number of studies using these mixed cultures have, however, produced contradictory results concerning their impact on wine quality. In fact, the inoculation procedure, as well as the different strains used, drastically impact the population dynamics of both species, thus modifying aroma production (Renault et al., 2015).

Few researchers have investigated volatile thiol formation by the *T. delbrueckii* yeast metabolism in pure and mixed cultures. According to Zott et al. (2011), in synthetic medium, *T. delbrueckii* released significant concentrations of 3SH but lower than that of pure *S. cerevisiae* cultures. It also has a poor capacity to form 4MSP. These results were confirmed by Sadoudi et al. (2012) during AF of Sauvignon Blanc must. As a result, in a simultaneous mixed culture, at a 10:1 ratio (*T. delbrueckii/S. cerevisiae)*, a decrease in 3SH and 3SHA production was observed, compared to a pure *S. cerevisiae* culture.

This study compared the volatile thiol profiles of Sauvignon Blanc wines fermented with pure *S. cerevisiae* and *T. delbrueckii*, as well as mixed cultures (simultaneous and sequential). To elucidate the nature of the interactions between these two yeast strains, 3SH, 3SHA, their related precursors, and 4MSP, as well as the population dynamics, were monitored throughout AF.

## Materials and Methods

### Yeast Strains

In this study, two commercial strains from Laffort company (France) were used: *S. cerevisiae* Zymaflore^®^ X5 and *T. delbrueckii* Zymaflore^®^ Alpha^TDn.sacch^. Yeasts were grown at 24°C on complete YPDA medium (1% yeast extract, 1% peptone, 2% dextrose) solidified with 2% agar, adjusted to pH 4.8.

### Fermentation Medium

The medium used was a Sauvignon Blanc grape must from Bordeaux area, pH: 3.15, with a sugar concentration of 203 g/L and an available nitrogen concentration adjusted to 210 mg/L (i.e., amino acids: 114 mg/L and ammonia: 96 mg/L). The total and free sulfur dioxide concentrations were, respectively, 60 and 19 mg/L. Before yeast inoculation, the must was sterilized by filtration (0.45 μm nitrate cellulose membrane, Millipore, Molsheim, France).

### Fermentation Conditions

Fermentation kinetics were monitored by CO_2_ release (Bely et al., 1990a,b). The amount of CO_2_ release (g/L) was determined by automatic measurement of fermentor weight loss every 20 min. The CO_2_ production rate (g/L/h) was obtained by polynomial smoothing of the last 11 CO_2_ measurements. The large number of CO_2_ acquisitions combined with precision weighing (0.01 g) gave three kinetic parameters with good accuracy: (1) lag phase (h) was the time between inoculation and the beginning of CO_2_ release, (2) *V*max (g/L/h) was the maximum CO_2_ production rate, and (3) FD (h) was the time required to ferment all the sugars in the medium. Weight loss due to evaporation was under 2%.

Yeasts were pre-cultured in Erlenmeyer flasks filled with must at 24°C for 24 h (*S. cerevisiae*) or 48 h (*T. delbrueckii*). Fermentations were carried out at 24°C with agitation in 1.2 L fermenters locked to maintain anaerobiosis throughout AF (CO_2_ was released through a sterile air outlet condenser). Four different trials were carried out: two pure cultures and two mixed cultures. Two types of mixed cultures were carried out: simultaneous mixed culture (called “simultaneous culture”) where *T. delbrueckii* and *S. cerevisiae* were inoculated at the same time and sequential mixed culture (called “sequential culture”) where *T. delbrueckii* was inoculated 24 h before *S. cerevisiae* yeast. Single and mixed cultures were inoculated with 1 × 10^7^ viable cells/mL for *T. delbrueckii* and 2 × 10^6^ viable cells/mL for *S. cerevisiae*. All experiments were performed in triplicate.

### Population Kinetics

In mixed cultures, yeast growth was determined by plate counting on two different agar media. Samples were withdrawn throughout fermentation and diluted appropriately. Non-*Saccharomyces* cells were counted using a specific agar medium (NS): YPDA (1% yeast extract, 1% peptone, 2% dextrose, 2% agar; pH 4.8) supplemented with 1 μg/mL cycloheximide to promote the growth of *T. delbrueckii* and inhibit that of *S. cerevisiae*. This low concentration allowed the growth of *T. delbrueckii* Zymaflore^®^ Alpha^TDn.sacch^ but inhibited that of *S. cerevisiae* Zymaflore^®^ X5 (data not shown). The number of *S. cerevisiae* was given as the difference between the total plate count using YPDA medium and the plate count using NS medium. Yeast growth in single cultures was determined using only the YPDA medium. At the end of AF, we controlled the species by PCR RFLP analysis of rDNA ITS region with digestion by Eco R1 (Granchi et al., 1999). Plates were incubated at 24°C for 4 days before counting.

### Wine Analysis

Ethanol concentration (% vol) was measured by infrared refractance (Spectra Analyser, Axflow, Plaisir, France) and sugar (g/L) was determined chemically by colorimetry (460 nm) in continuous flux (Sanimat, Montauban, France). These analyses were performed by Sarco laboratory (Bordeaux, France).

### Volatile Thiols Analysis

Volatile thiol quantification was performed by the wine analysis laboratory Sarco (Bordeaux, France). 4MSP, 3SH, and 3SHA were specifically extracted by reversible combination of the thiols with sodium-*p*-hydroxymercuribenzoate (*p*-HMB), from 50 mL wine previously preserved from oxidation by adding 50 mg/L of SO_2_, as described by Tominaga and Dubourdieu (2006) and quantified by gas chromatography–mass spectrometry according to methods described by Tominaga et al. (1998a) and Tominaga and Dubourdieu (2000).

### 3SH Thiol Precursors Analysis

Cys-3SH and Glut-3SH were assayed according to the protocol described by Capone et al. (2010), modified as follows. An aliquot (25 μL) of an aqueous solution containing *d_3_*-Glut-3SH (final concentration 50 μg/L) was added to 1 mL grape juice. The sample was diluted with 2 mL water and passed through a 6 mL, 500 mg LC-18 cartridge (Supelco), previously conditioned with 4 mL methanol, followed by 2 mL methanol-water (50/50) mix, and 3 mL water. After loading the juice, the sorbent was rinsed with 1 mL water, dried under air for 1 min, and eluted with 3 mL methanol solution (70%). The eluate was collected and dried in a Vacuum System with Vortex Motion (RapidVap, Labconco, US) at 10 mbar and 45°C. The extract was diluted in formic acid solution (700 μL, 0.1%), filtered through a 0.45 μm filter for LC–MS analysis. All LC–MS analyses were carried out on an Accela UHPLC (Thermo Fisher Scientific), connected in series to an Exactive (Thermo Fisher Scientific, Bremen, Germany) mass spectrometer, equipped with a heated ESI ion source. The column was a 100 × 2.1 mm, i.d., 1.7 μm, Synchronis aQ (Thermo Scientific). The solvents were: 0.1% aqueous formic acid (solvent A) and 0.1% formic acid in acetonitrile (solvent B), with a flow rate of 300 μL/min. The gradient for solvent B was as follows: 0 min, 9%; 0.8 min, 9%; 5 min, 40%; 5.2 min, 90%. The column was equilibrated with 9% B for 1 min prior to injection. A 5 μL injection volume was used for each sample. The ion source was operated in the positive ion mode at 3.5 kV. Source vaporizer temperature was set at 300°C, capillary temperature at 350°C, nitrogen sheath gas at 80, and the auxiliary and sweep gas at 5 (arbitrary units). A mass range of 100–500 was acquired in full scan MS mode. The resolution setting was 25 000 (m/Δm, fwhm at m/z400).

### Statistical Analysis

In order to compare modalities, data were analyzed by single-factor variance (ANOVA, *p* < 0.05), following verification of variance homogeneity (Levene test, *p* > 0.05). Thereafter, a multiple comparison test (Duncan) was applied to classify the different culture protocols (*p* < 0.05). All statistics were analyzed using the R program.

## Results and Discussion

### CO_2_ Release and Population Kinetics in Pure and Mixed Cultures

Four different AF were conducted in Sauvignon Blanc grape must: two with pure cultures and two with mixed cultures (inoculated either simultaneously or sequentially). In all trials, the must was inoculated with 1 × 10^7^ viable cells/mL for *T. delbrueckii* and 2 × 10^6^ viable cells/mL for *S. cerevisiae.* The final ethanol concentrations (12% vol, corresponding to a final CO_2_ release of 97 g/L) were reached in all fermentations except in the pure *T. delbrueckii* culture, which predictably stopped fermenting at 6.2%vol.

**FIGURE 1:**
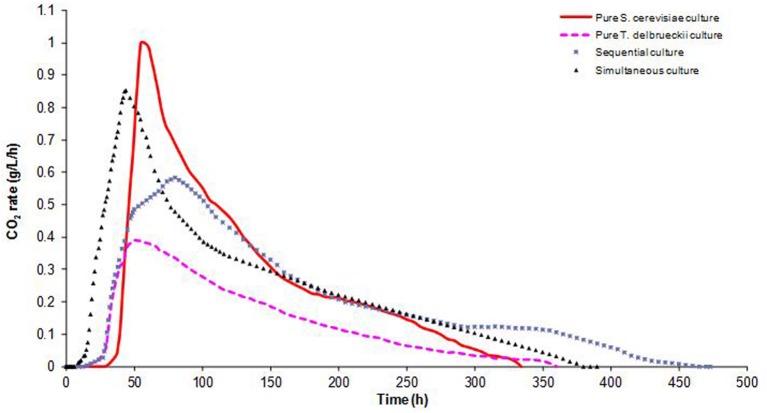
**CO_2_ production rates (g/L/h) over time in pure and mixed *T. delbrueckii/S. cerevisiae* cultures.** Average values of three experiments, standard deviation <5%.

The overall fermentation kinetic profiles, i.e., the variation in CO_2_ rate versus time, are shown in **Figure 1**. The rate curves varied markedly from one culture to another. Indeed, the trial involving inoculation with *T. delbrueckii* alone showed a short lag phase (17 h), but also a low fermentation rate, characterized by the lowest *V*max (0.39 g/L/h). In contrast, even with a long lag phase (34 h), the *S. cerevisiae* culture had a high fermentation rate with the highest *V*max (1 g/L/h) and the shortest fermentation duration (334 h). These results are in good agreement with previous investigations using a large number of strains (Renault et al., 2009), where *T. delbrueckii* was found to have a lower fermentation capacity than *S. cerevisiae*.

Mixed cultures exhibited intermediate fermentation kinetics (**Figure 1**). When both species were added at the same time, the fermentation curve showed similar profiles to that of pure *S. cerevisiae* culture, but with a lower *V*max (0.84 g/L/h) and a shorter lag phase time (11 h), due to the larger amount of cells inoculated (1.2 × 10^7^ viable cells/mL). On the contrary, when *T. delbrueckii* and *S. cerevisiae* were inoculated sequentially, the fermentation curve was close to that of *T. delbrueckii* alone, except that the *V*max was higher (0.56 g/L/h). The fermentation of the sequential culture took longer than that of the simultaneous or pure *S. cerevisiae* cultures.

**FIGURE 2:**
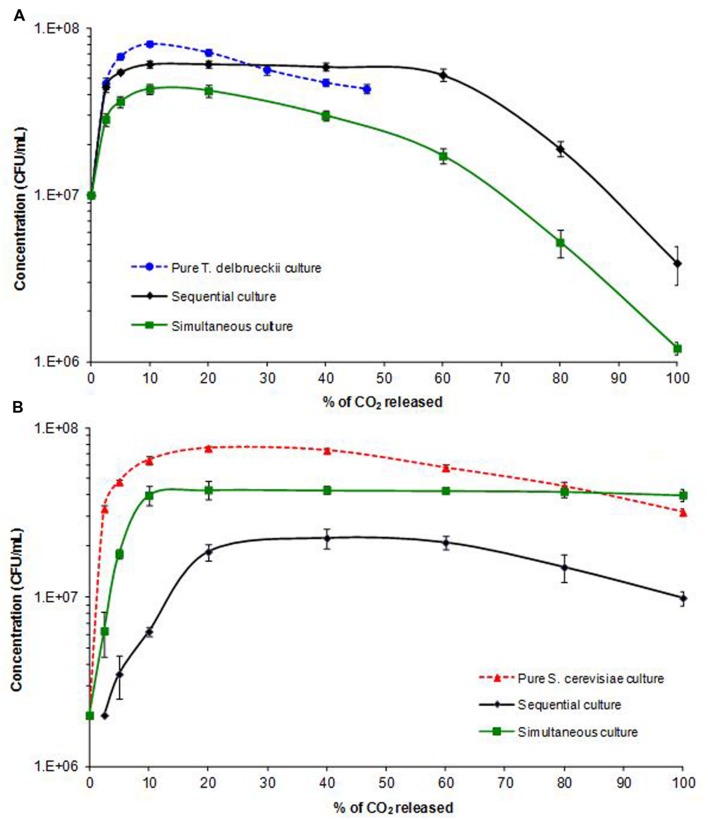
**Kinetics of *T. delbrueckii***(A)** and *S. cerevisiae***(B)** cell populations (CFU/mL) during alcoholic fermentation (AF) in pure and mixed cultures.** Average value of three experiments.

The viable *S. cerevisiae* and *T. delbrueckii* populations in pure and mixed cultures were determined by plate counting (**Table 1**). The biomass kinetics are presented according to AF progress (expressed in % of CO_2_ released; **Figure 2**). The maximum population (*X*max) reached during AF by *T. delbrueckii* and *S. cerevisiae* was higher when they were inoculated alone (8.1 × 10^7^ viable cells/mL for *T. delbrueckii* and 7.6 × 10^7^ viable cells/mL for *S. cerevisiae*, in pure cultures, respectively) than in sequential and simultaneous cultures. Hence, both species influenced each other’s development. It is noteworthy that the *X*max of *T. delbrueckii* in the sequential culture was higher (6.1 × 10^7^ viable cells/mL) than in the simultaneous culture (4.3 × 10^7^ viable cells/mL). On the contrary, the *X*max of *S. cerevisiae* was 4.4 × 10^7^ and 2.4 × 10^7^ viable cells/mL, in simultaneous and sequential cultures, respectively.

**Table 1:** Maximal cell population and final volatile thiol concentrations in pure and mixed *T. delbrueckii* and *S. cerevisiae* cultures.

	*Torulaspora delbrueckii* pure culture	Sequential mixed culture	Simultaneous mixed culture	*Saccharomyces cerevisiae* pure culture
**Maximal population (viable cells/mL)**				
*T. delbrueckii*	8.1 × 10^7^ ± 2.8 × 10^6c^	6.1 × 10^7^ ± 7.1 × 10^6b^	4.3 × 10^7^ ± 3.5 × 10^6a^	/
*S. cerevisiae*	/	2.4 × 10^7^ ± 7.1 × 10^5a^	4.4 × 10^7^ ± 2.3 × 10^6b^	7.6 × 10^7^ ± 1.8 × 10^6c^
**Final volatile thiol concentrations (ng/L)**				
3SH	623 ± 103^b^	1312 ± 224^c^	362 ± 97^a^	303 ± 141^a^
3SHA	14 ± 2^a^	218 ± 34^c^	79 ± 20^b^	83 ± 9^b^


*Average values of three experiments standard deviation. Represents significantly different statistical groups (*p* 0.05). 3SH, 3-sulfanylhexan-1-ol; 3SHA, 3-sulfanylhexyl acetate.*

Indeed, in sequential culture, when the addition of *S. cerevisiae* (when 2.5 g/L CO_2_ had been released) was delayed, *T. delbrueckii* was able to grow from 1 × 10^7^ viable cells/mL to 4.1 × 10^7^ viable cells/mL within the first 24 h, thus initiating AF (**Figure 2**). In that case, the *T. delbrueckii/S. cerevisiae* ratio after 24 h was largely in favor of *T. delbrueckii* (about 20:1) but *S. cerevisiae* developed sufficiently (from 2 × 10^6^ to 2.4 × 10^7^ viable cells/mL) to complete AF.

Consequently, in the sequential culture, the *X*max of *T. delbrueckii* was maintained during the first 60% of AF and its viable population was higher than that of *S. cerevisiae* during the first 85% of AF (**Figure 2**). The dominance of *T. delbrueckii* throughout the AF, in sequential culture, is probably due to higher consumption of dissolved oxygen, nitrogen and vitamins than *S. cerevisiae* which was inoculated 24 h after.

The kinetics of the two yeast populations were very different following simultaneous inoculation, where the initial inoculation ratio of 5:1 (1 × 10^7^ viable cells/mL *T. delbrueckii* and 2 × 10^6^ viable cells/mL *S. cerevisiae*) was less favorable to *T. delbrueckii*, which was only dominant during the first 10% of AF. Indeed, the *X*max of *T. delbrueckii* in simultaneous inoculation was lower than in sequential culture (4.3 × 10^7^ and 6.1 × 10^7^ viable cells/mL, respectively). Furthermore, *S. cerevisiae* also reached its *X*max during the early stage of AF and maintained this level of population until the end of AF, whereas the viable *T. delbrueckii* population decreased rapidly after 10% of AF. According to several authors (Nissen and Arneborg, 2003; Nissen et al., 2003; Renault et al., 2013), the physical contact/proximity between *T. delbrueckii* and a large viable population of *S. cerevisiae* induced the rapid death of *T. delbrueckii*. Competition for oxygen may also explain the rapid death of *T. delbrueckii* cells (Hansen et al., 2001). Indeed, while *S. cerevisiae* yeast is able to grow rapidly under strictly anaerobic conditions, *T. delbrueckii* is affected by a lack of oxygen (Hanl et al., 2005).

To sum up, sequential culture facilitated the development of *T. delbrueckii*, resulting in a larger viable population than that of *S. cerevisiae* almost until the end of AF. Under these conditions, the kinetic parameters were close to those obtained in pure *T. delbrueckii* culture, except that AF was completed. In contrast, when both species were inoculated simultaneously, the maximal viable populations of both species were similar, but that of *S. cerevisiae* was larger than that of *T. delbrueckii* during 90% of the reaction, with AF showing similar profiles to those of pure *S. cerevisiae* cultures. Nevertheless, *T. delbrueckii* had a small impact on fermentation kinetics, as *V*max was lower and AF was extended, in comparison to the pure *S. cerevisiae* culture.

### Volatile Thiol Production

*Torulaspora delbrueckii* in pure culture did not produce 4MSP, unlike *S. cerevisiae* (33 ng/L at the end of AF). Very small amounts were detected in mixed cultures (<7 ng/L), suggesting the absence of any interaction between the species in producing this compound. These results confirmed the inability of *T. delbrueckii* to release 4MSP, as already reported in previous studies (Zott et al., 2011; Sadoudi et al., 2012).

**FIGURE 3:**
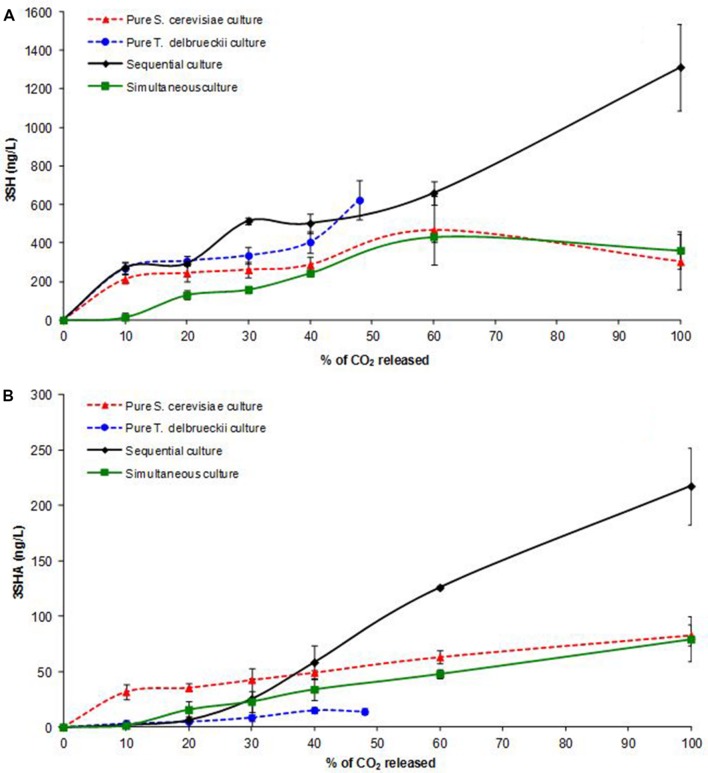
**Kinetics of 3SH **(A)** and 3SHA **(B)** concentrations (ng/L) in pure and mixed *T. delbrueckii/S. cerevisiae* cultures during AF.** Average value of three experiments.

As shown **Figure 3**, 3SH production was similar in both pure cultures during the first 20% of AF but diverged after this point, with differences in the final concentrations. Indeed, at the end of fermentation, the 3SH concentration in the pure *T. delbrueckii* culture was twofold higher than that in the pure *S. cerevisiae* culture (623 and 303 ng/L, respectively; **Table 1**). This result differed from previous findings using other *T. delbrueckii* strains (Zott et al., 2011; Sadoudi et al., 2012), suggesting that this production is strain-dependent.

Results were different for 3SHA for which *T. delbrueckii* produced very small amounts (14 ng/L), showing a progressive and linear production throughout AF (**Figure 3**). On the contrary, total 3SHA production by *S. cerevisiae* was higher (83 μg/L), with 50% occurring during the growth phase. Furthermore, the 3SH/3SHA ratios were 3.6 and 44.5 in pure *S. cerevisiae* and *T. delbrueckii* cultures, respectively. These results confirmed those obtained by Sadoudi et al. (2012), showing that *T. delbrueckii* had a lower acetylation activity (i.e., a low conversion rate of 3SH to 3SHA) than *S. cerevisiae* (Coetzee and du Toit, 2012).

Furthermore, no significant difference in 3SH and 3SHA production was observed between the simultaneous and pure *S. cerevisiae* cultures at the end of AF (**Table 1**). However, it is interesting to note that, in the simultaneous inoculation protocol, the beginning of production was delayed (no production during the first 10% of AF; **Figure 3**).

Concerning the sequentially inoculated culture, 3SH production was similar to that of the pure *S. cerevisiae* culture until 20% of AF, but diverged beyond that point, exhibiting a major increase during the last stage in AF, resulting in significantly different final concentrations. Indeed, at the end of AF, the 3SH concentration in the sequential culture was fourfold higher than in the pure *S. cerevisiae* culture (1312 and 303 ng/L, respectively; **Table 1**). 3SHA production in sequential culture was also different from the pure *S. cerevisiae* culture, remaining very weak until 20% of AF and then drastically increasing to reach a final concentration nearly threefold higher than in the pure *S. cerevisiae* culture (218 and 83 ng/L, respectively; **Figure 3**).

These results suggested that sequential inoculation of *S. cerevisiae* and *T. delbrueckii* in Sauvignon Blanc must resulted in synergistic interactions that affected 3SH and 3SHA production during AF.

### Volatile Thiol Precursors

**FIGURE 4:**
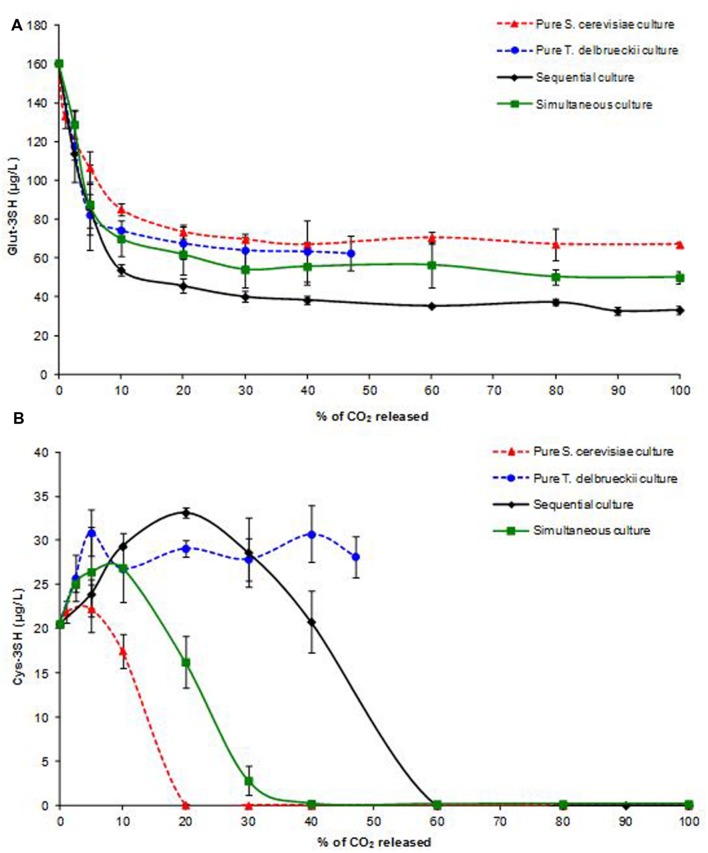
**Kinetics of Glut-3SH **(A)** and Cys-3SH **(B)** concentrations (μg/L) in pure and mixed *T. delbrueckii/S. cerevisiae* cultures during AF.** Average value of three experiments.

To investigate the possible synergistic interactions between the two species resulting in higher concentrations of 3SH and its acetate at the end of AF, their *S*-conjugate precursors (Cys-3SH and Glut-3SH) were monitored throughout fermentation (**Figure 4**). Cys-3SH and Glut-3SH were detected (0% of AF) at normal levels for a Sauvignon Blanc must: 20 μg/L and 160 μg/L, respectively.

In pure *S. cerevisiae* and *T. delbrueckii* cultures, only 60% of the initial Glut-3SH concentration was assimilated, leaving approximately 65 μg/L of the precursor in the wine. However, in simultaneous and sequential cultures uptake was significantly higher. Indeed, up to 69 and 79% of Glut-3SH were assimilated, resulting in wines with 50 and 33 μg/L precursor, respectively.

**Figure 4** shows that, for all modalities, Glut-3SH was rapidly metabolized by the yeast in the earliest stage of AF, immediately after yeast addition. In the pure *S. cerevisiae* culture, the uptake stopped at 20% of AF. In all cultures involving *T. delbrueckii*, Glut-3SH kinetics were similar until 5% of AF, then the assimilation slowed down suddenly and stopped around 20% of AF in pure *T. delbrueckii* and simultaneous cultures. In the sequential culture, Glut-3SH assimilation continued slowly after 20% of AF, resulting in the lowest final Glut-3SH concentration in the medium. This enhanced precursor assimilation may explain the variations in total 3SH and 3SHA release observed at end of AF.

The kinetics of cysteine *S*-conjugate precursor (Cys-3SH) concentrations during AF is probably due to the fact that it is both produced and assimilated by the yeast (Cordente et al., 2015). Cys-3SH kinetics varied markedly from one culture to another (**Figure 4**). In the pure *S. cerevisiae* culture, Cys-3SH concentrations decreased after a short lag phase (<5% of AF) and completely disappeared after 20% of AF. In contrast, in all cultures involving *T. delbrueckii*, an increase in Cys-3SH concentrations was observed in the early stage of AF (**Figure 4**). In the pure *T. delbrueckii* culture, the concentration increased by 50% (21 μg/L initial to 32 μg/L at 5% of AF) during the very early stages of AF. In this phase, Cys-3SH production was concomitant to Glut-3SH depletion (**Figure 4**) and 3SH release (**Figure 3**), suggesting that *T. delbrueckii* was able to synthesize Cys-3SH and 3SH from Glut-3SH, but the cysteinylated form was hardly assimilated, if at all, by the yeast. In *S. cerevisiae*, its transport is provided by the Gap1p membrane protein (Subileau et al., 2008a). The *GAP1* gene has not been clearly identified in the genome sequence of *T. delbrueckii*, type strain CBS 1146T (CLIB230T). Indeed, the closest BLAST of Gap1p protein from *S. cerevisiae* S288c against CBS 1146T is a hypothetical protein (TDEL_0C00930) with only 74% identity along 96% of the sequence. It was, therefore, hypothesized that Gap1p permease was absent or dysfunctional in this species. Further experiments are required to validate this hypothesis, for example, an intracellular Cys-3SH assay.

When Glut-3SH stopped being converted into Cys-3SH, its concentration in the medium remained constant until the end of AF.

Interestingly, throughout AF in the two mixed cultures, the more *T. delbrueckii* developed, the higher the Cys-3SH concentration became. In the sequential culture, where *T. delbrueckii* dominated *S. cerevisiae* for 85% of AF (**Figure 2**), the Cys-3SH accumulation phase in the must was much longer than in the simultaneous culture, resulting in a higher concentration at the end of this phase (34 μg/L instead of 27 μg/L). After this accumulation phase, in both mixed cultures, an abrupt depletion reduced Cys-3SH to undetectable levels in the medium after around 40 and 60% of AF, in simultaneous and sequential cultures, respectively. It is worth noting that Cys-3SH uptake was apparently correlated with the development of *S. cerevisiae* (**Figure 2**). Precursor uptake began when *S. cerevisiae* reached its *X*max, around 10 and 20% of AF (corresponding to 4.3 × 10^7^ and 2.4 × 10^7^ viable cells/mL in simultaneous and sequential cultures, respectively). Moreover, the Cys-3SH degradation rate (i.e., the slope of the line) of both mixed culture was apparently correlated to *S. cerevisiae X*max level. The steep slope observed for the pure *S cerevisiae* culture, with *X*max around 7.5 × 10^7^ viable cells/mL, supports this hypothesis.

## Conclusion

This study, based on an analysis of the main thiols throughout AF of Sauvignon Blanc must fermented by *T. delbrueckii* and *S. cerevisiae* in pure or mixed cultures, provides interesting insights into the metabolic pathway of thiols in *T. delbrueckii* and reveals a synergistic interaction between the two species.

Under these experimental conditions, *T. delbrueckii* produced no 4MSP and only very small amounts of 3SHA, confirming previous findings. In contrast, high 3SH levels were found in wines fermented with pure *T. delbrueckii* and sequential *T. delbrueckii/S. cerevisiae* cultures, in comparison to wines resulting from AF with only *S. cerevisiae*. Monitoring 3SH and its precursors (Glut-3SH and Cys-3SH) throughout AF led us to conclude that *T. delbrueckii* only assimilates the glutathionylated precursor, while both precursor forms are metabolized by *S. cerevisiae.* In pure *T. delbrueckii* cultures, Glut-3SH degradation produced significant amounts of 3SH and Cys-3SH in the wine. In mixed cultures, the more *T. delbrueckii* developed, the higher the Glut-3SH uptake and Cys-3SH release. In sequential cultures, which favored *T. delbrueckii* development compared to the simultaneous protocol, the results revealed an increase in the cysteinylated precursor followed by an increase in 3SH. Hence, once released by *T. delbrueckii*, the cysteinylated precursor was converted into 3SH by *S. cerevisiae* in the last stage of AF. The direct consequence was higher overall 3SH and 3SHA production than in pure *S. cerevisiae* cultures. Further work with different strains of *T. delbrueckii* and *S. cerevisiae* will help to confirm the synergistic interaction described between these two species.

## Author Contributions

Conceived and designed the experiments: JC, VM, CT, MB, and PR. Performed the experiments: PR and CT.

## Conflict of Interest Statement

The authors declare that the research was conducted in the absence of any commercial or financial relationships that could be construed as a potential conflict of interest.
